# Traumatic J-Pouch Perforation following a Blunt Abdominal Injury

**DOI:** 10.1155/2021/6686964

**Published:** 2021-07-13

**Authors:** Leonid Drober, David Hochstein, Hany Bahouth

**Affiliations:** Department of General Surgery, Rambam Medical Center and the Ruth and Bruce Rappaport Faculty of Medicine, Technion–Institute of Technology, Haifa, Israel

## Abstract

A 46-year-old male was admitted to the trauma department after a motor vehicle accident. He presented with severe abdominal pain and a distended abdomen with peritonitis. His past surgical history included total proctocolectomy with ileal J-pouch anal anastomosis for ulcerative colitis 20 years previously. Computed tomography showed free peritoneal air and fluid in the abdomen mandating an exploratory laparotomy. A perforation at the ileal J-pouch blind end was found. Primary closure with diverting loop ileostomy was performed. The patient had an uneventful recovery and underwent closure of the ileostomy two months later. The case and management are discussed after reviewing the literature.

## 1. Introduction

Restorative proctocolectomy with ileal J-pouch anal anastomosis has become a standard operation for ulcerative colitis in consideration of the patient's quality of life. Although the long-term outcome of this procedure is generally satisfactory [1], occasional postsurgical complications have been reported [2–4]. Ileal pouch perforation is one of these rare complications [5–9]. We report a rare trauma case of ileal J-pouch perforation at the blind end after a severe motor vehicle accident.

## 2. Case Report

A 46-year-old male was admitted to the trauma shock room due to his involvement in a motor vehicle accident. He presented with severe abdominal pain and a distended abdomen with peritoneal signs. The patient underwent a total proctocolectomy with ileal J-pouch anal anastomosis for ulcerative colitis, 20 years previously. He also suffered several events of pouchitis.

Laboratory workup on admission revealed a white blood cell count of 12,500/mcL (4000-10,800), an arterial blood gas analysis lactate level of 4.1 mmol/L (0-1.3). Abdominal computed tomography with IV contrast injection but no oral or rectal contrast fluid added, in accordance to trauma imaging protocol, showed free intraperitoneal air and fluid.

The patient underwent emergency exploratory laparotomy on admission, which revealed a large amount of turbid ascites in the abdominal cavity and peritoneal adhesions. After careful adhesiolysis, we did not identify the perforation site. We injected methylene blue dye through the nasogastric tube, which did not reveal perforation of the stomach or duodenum.

**Figure 1:**
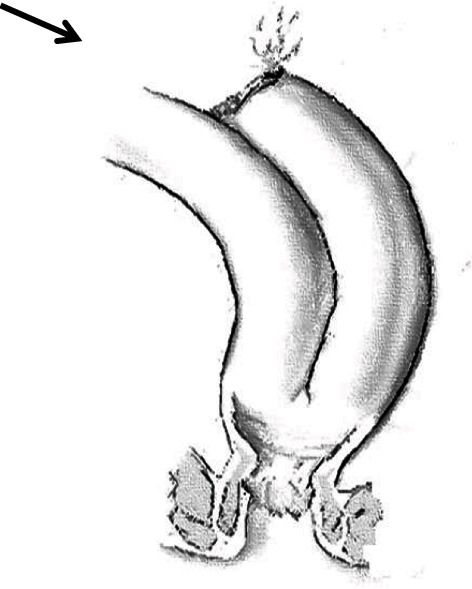
Perforation at the blind end of ileal J-pouch.

We then injected air rectally after soaking the pelvis in sterile water and witnessed a “positive bubble test,” revealing a leak of air and feces through a 10 mm perforation at the blind end of the J-pouch (Figure 1). There were no signs of direct trauma to the pelvis, rectum, or surrounding tissues to explain this finding. We concluded that the mechanism of injury was an acute expansion of the pouch and perforation of the blind end as a point of weakness at the staple line. We opted to perform a two-layer primary closure of the perforation with absorbable sutures without resection of any part of the pouch. Since the above mechanism was not a direct perforation, but a “blowout” mechanism, we fashioned a protecting diverting loop distal ileostomy. Two months later, the ileostomy was closed with an uneventful operative and postoperative course.

## 3. Discussion

Traumatic perforation of the ileal pouch is rare. We found only one case report in the literature of ileal pouch perforation [7], caused by blunt trauma to the lower abdomen.

The intestine is the third most commonly injured abdominal organ in blunt trauma. Minor injury may result in only a serosal tear or mild contusion of the intestine. Major injury may cause transmural perforation or transection of the bowel, mesenteric injury resulting in ischemic bowel, contusion of the bowel wall with seromuscular damage, or injury to the root of the mesentery, resulting in a tear of the mesentery or vessels. The mechanism of the injury has been suggested by Moty [10] as follows: (1) a crush injury between the vertebrae and anterior abdominal wall, (2) a sudden increase in intraluminal pressure in the bowel, or (3) tangential tears at relatively fixed points along the bowel.

In patients with restorative *proctocolectomy*, the pouch was brought down to the anal area for anastomosis. Thus, the vascular pedicle to the pouch is under much more tension and is fixed [11, 12]. This may predispose to vasculature injury after blunt trauma. Also, a distended pouch may resemble a distended bladder and is known to be more prone to injury after blunt trauma. A pouch is probably more easily perforated when it is distended, inflamed, or strictured distally. Mortality rate for intestinal injury ranges from 10 to 30%, and missed or delayed diagnoses is a contributing factors for higher mortality [10], and when facing a trauma patient in peritonitis who has an ileal pouch, it is mandatory to rule out perforation of the pouch. Bacterial overgrowth in the pouch is common [9, 13, 14]. Perforation of an ileal pouch may result in much more severe peritonitis as encountered in colonic perforation.

To decrease the morbidity after perforation of a pouch, the following guidelines should be employed: (1) a high index of suspicion is essential; (2) a diagnostic aid (such as chest X-ray, CT scan (preferably with transanal contrast), and peritoneal lavage) should be used properly; (3) antibiotics for aerobic and anaerobic bacteria should be given; (4) early surgery is mandatory; (5) appropriate surgical drainage is important for the outcome. With limited experience, it remains however difficult to draw conclusions as to whether proximal diversion of the ileostomy is necessary.

In our injured patient, pouch perforation occurred after traumatic seat belt injury.

Transmission of the trauma forces to a distended, possibly inflamed ileal pouch is probably the main reason for perforation.

In conclusion, we report a rare case of perforation in the blind end of the ileal J-pouch occurring after a severe seat belt injury. We present our thought process and diagnostic workup. It is mandatory to rule out a blow out of an ileal pouch after a severe abdominal trauma if there is evidence of perforation of hollow viscus viscera.
